# Interventions to Reduce Medication Dispensing, Administration, and Monitoring Errors in Pediatric Professional Healthcare Settings: A Systematic Review

**DOI:** 10.3389/fped.2021.633064

**Published:** 2021-05-26

**Authors:** Joachim A. Koeck, Nicola J. Young, Udo Kontny, Thorsten Orlikowsky, Dirk Bassler, Albrecht Eisert

**Affiliations:** ^1^Hospital Pharmacy, Rheinisch-Westfälische Technische Hochschule Aachen University Hospital, Aachen, Germany; ^2^Section of Pediatric Hematology, Department of Pediatric and Adolescent Medicine, Rheinisch-Westfälische Technische Hochschule Aachen University Hospital, Aachen, Germany; ^3^Section of Neonatology, Department of Pediatric and Adolescent Medicine, Rheinisch-Westfälische Technische Hochschule Aachen University Hospital, Aachen, Germany; ^4^Department of Neonatology, University Hospital Zurich, Zurich, Switzerland; ^5^Institute of Clinical Pharmacology, University Hospital of Rheinisch-Westfälische Technische Hochschule Aachen, Aachen, Germany

**Keywords:** medication error, medication safety, child, pediatric, dispensing error, administration error, monitoring error, hierarchy of controls

## Abstract

**Introduction:** Pediatric patients cared for in professional healthcare settings are at high risk of medication errors. Interventions to improve patient safety often focus on prescribing; however, the subsequent stages in the medication use process (dispensing, drug administration, and monitoring) are also error-prone. This systematic review aims to identify and analyze interventions to reduce dispensing, drug administration, and monitoring errors in professional pediatric healthcare settings.

**Methods:** Four databases were searched for experimental studies with separate control and intervention groups, published in English between 2011 and 2019. Interventions were classified for the first time in pediatric medication safety according to the “hierarchy of controls” model, which predicts that interventions at higher levels are more likely to bring about change. Higher-level interventions aim to reduce risks through elimination, substitution, or engineering controls. Examples of these include the introduction of smart pumps instead of standard pumps (a substitution control) and the introduction of mandatory barcode scanning for drug administration (an engineering control). Administrative controls such as guidelines, warning signs, and educational approaches are lower on the hierarchy and therefore predicted by this model to be less likely to be successful.

**Results:** Twenty studies met the inclusion criteria, including 1 study of dispensing errors, 7 studies of drug administration errors, and 12 studies targeting multiple steps of the medication use process. A total of 44 interventions were identified. Eleven of these were considered higher-level controls (four substitution and seven engineering controls). The majority of interventions (*n* = 33) were considered “administrative controls” indicating a potential reliance on these measures. Studies that implemented higher-level controls were observed to be more likely to reduce errors, confirming that the hierarchy of controls model may be useful in this setting. Heterogeneous study methods, definitions, and outcome measures meant that a meta-analysis was not appropriate.

**Conclusions:** When designing interventions to reduce pediatric dispensing, drug administration, and monitoring errors, the hierarchy of controls model should be considered, with a focus placed on the introduction of higher-level controls, which may be more likely to reduce errors than the administrative controls often seen in practice. Trial Registration Prospero Identifier: CRD42016047127.

## Introduction

The ongoing need to address medication safety and reduce the risks to patients from their medications was recently reinforced by the World Health Organization Technical Report, “Medication Safety in High-Risk Situations”; the report highlighted that adverse events resulting from medication errors are now estimated to be the 14th leading cause of morbidity and mortality in the world (1). In pediatric patients, pharmacokinetic and pharmacodynamic parameters can be significantly different from those in adults (2). As pediatric specific formulations are often not available, adult formulations have to be manipulated off-label before use (e.g., through crushing a tablet and taking a portion from it). This results in an additional risk for miscalculations (2). As a result, children are at an estimated three times higher risk of potential adverse drug events than adults (3).

Medication errors are broadly defined as “any preventable event that may cause or lead to inappropriate medication use or patient harm while the medication is in the control of the healthcare professional, patient, or consumer” (4). These events may occur in every step of the medication use process (MUP). Aitken et al. describe the MUP as four stages, starting with prescribing, followed by preparation and/or dispensing of the medication, and then drug administration and finally monitoring for both therapeutic and adverse effects (5). The MUP is cyclical, and depending on the outcomes of the monitoring process, the decision may be made to either stop a medication or issue a further prescription for the same or a different medication. It has been suggested that the drug administration step of the MUP is the most prone to error (5). Despite this, the current literature focuses on interventions to prevent and/or reduce prescribing errors, with a lesser focus on the subsequent stages of the MUP. As the last overview of these interventions was published in 2014 (incorporating data up to November 22, 2011) (6), a follow-on is needed. This review seeks to identify interventions designed to reduce and/or prevent drug dispensing, administration, and monitoring errors and determine their effect.

## Methods

### Protocol and Registration

The protocol was registered in PROSPERO (reg. no. CRD42016047127) and with the local ethics commission (EK 158/17). To promote a differentiated focus on the individual stages of the MUP, the results of the review were split *post-hoc*, to consider interventions targeting prescribing errors in another publication. The structure of this article was guided by the recommendations of Preferred Reporting Items for Systematic Reviews and Meta-Analyses (PRISMA) (7).

### Eligibility Criteria

Studies of interventions to reduce drug dispensing, administration, and/or monitoring errors in professional pediatric healthcare settings were included. An intervention was defined as any action or set of actions implemented with the aim of reducing medication errors. The definition of a medication error from the “National Coordinating Council for Medication Error Reporting and Prevention” was adopted, to provide a broad classification and allow for the inclusion of as many studies as possible (4). Definitions of dispensing errors, drug administration errors, and monitoring errors adopted for this review are provided in Table 1 along with their sources (8–10). A professional healthcare setting was defined as all inpatient and outpatient facilities where a healthcare professional is involved in the MUP. Medication errors occurring when the medication is in the care of the patient and/or their family are outside the scope of this systematic review.

**Table 1:** Definitions of error subtypes.

**Type of error**	**Definition**
Medication error (ME)	“A medication error is any preventable event that may cause or lead to inappropriate medication use or patient harm while the medication is in the control of the healthcare professional, patient, or consumer. Such events may be related to professional practice, healthcare products, procedures, and systems, including prescribing, order communication, product labeling, packaging, and nomenclature, compounding, dispensing, distribution, administration, education, monitoring, and use” (4).
Dispensing error (DE)	“Any unintended deviation from an interpretable written prescription or medication order. Both content and labeling errors are included. Any unintended deviation from professional or regulatory references, or guidelines affecting dispensing procedures, is also considered a dispensing error” (8).
Administration error (AE)	“Administration of a dose of medication that deviates from the prescription, as written on the patient medication chart, or from standard hospital policy and procedures. This includes errors in the preparation and administration of intravenous medicines on the ward” (9).
Monitoring error (MO)	“When a prescribed medicine is not monitored in the way that would be considered acceptable in routine general practice. It includes the absence of tests being carried out at the frequency listed in the criteria, with tolerance of +50%” (10). This includes monitoring after initiation and continuation of therapy.

Five study types were included. Definitions from the “Cochrane Effective Practice and Organization of Care Review Group” were adopted for “randomized controlled trial,” “controlled clinical trial,” “controlled before–after study,” and “interrupted time–series study.” The fifth study type included “uncontrolled before–after study,” defined as a study involving a comparison of two patient groups, with and without the investigated intervention.

Outcome parameters defined for the analysis were as follows: intervention types and their impact on reducing dispensing, drug administration, and/or monitoring errors according to each article's assessment.

### Information Sources

The search strategy was adapted from the previous systematic review by Rinke et al. (6) (see Supplementary Material 1).

### Search

The analysis included the time span from November 22, 2011, to December 31, 2019, in order to provide a follow-on from the previous systematic review (6). Previously piloted terms were used to search CENTRAL, CINAHL, EMBASE, and MEDLINE. Reference searching in the bibliographies of each included article and selected reviews (6, 11–51) complemented the aforementioned search.

### Study Selection

One reviewer (JK) assessed the resulting titles and abstracts using a piloted form adapted from the form initially used by Rinke et al. (6). Abstracts were evaluated according to 16 exclusion criteria (see Supplementary Table 1). If none of the criteria were met, the abstract was included in the screening of full texts against these criteria. A second reviewer (AE) independently examined a random 10% of the first reviewer's search results of each database using the same form. Interrater agreement was calculated via Cohen κ (52).

### Data Collection Process

Data extraction was performed using a piloted form, first by one (JK) and then by a second reviewer (AE). Results were discussed and amended if necessary. The results of the literature research were analyzed using Excel 2016 (Redmond, WA, USA).

### Data Items

Data collection included the interventions that were tested and their reported effect, the study type, number and characteristics of included patients, and the type of healthcare professionals delivering the intervention(s).

### Risk of Bias in Individual Studies

Bias risk was evaluated according to the COCHRANE tool for randomized controlled trials, controlled clinical trials, controlled before–after studies, and interrupted time–series studies (53, 54), whereas uncontrolled before–after studies were rated via ROBINS-I (55).

### Summary Measures

The impact of interventions was assessed through the calculation of the error rate (based on each author's definition of a medication error) for the control and intervention groups. Based on this, “absolute risk reduction” was established for each single intervention or bundle of interventions. When insufficient data were available to calculate separate error ratios for two study groups, this was not undertaken, and the ratio of error before and after intervention was calculated, based on the ratios given by individual study authors (Table 2).

**Table 2:** Summary of study characteristics.

**Studies addressing one step in the medication use process**									
**First author (country, year)**	**Error type(s)**	**Study design, study centers**	**Setting**	**Methods**	**Intervention**	**Single/bundle intervention**	**Results**	**Error rate CG/IG**	**Absolute risk reduction (reported level of significance)**
Campino et al. (Spain, 2016) (56)	Drug administration errors	UBA, multicenter	10 NICU and one hospital pharmacy	Assessment of randomly collected intravenous dilutions of vancomycin, gentamicin, phenobarbital, and caffeine citrate to investigate calculation and accuracy errors during preparation; most dilutions contained vancomycin or gentamicin (75% and 71%, respectively)	(1) Standardized preparation protocol with no need for calculation; (2) Educational program, developed by pharmacists, nurses, and physicians including preinterventional results, basic rules for medication preparation, weak points of medication preparation, and new preparation protocols; education was repeated several times	Bundle	Calculation errors: CG: 6, IG:0; all NICUs/hospital pharmacy benefited from the intervention (but to different degrees); total: 266 accuracy errors (underdosing/overdosing)/504 preparations, IG: 67/332	52.78%/21.99%	30.79% (significantly positive results)
Chedoe et al. (Netherlands, 2012) (57)	Drug administration errors	UBA, single center	NICU	Direct observation of ward-based preparation and application of drugs by trained pharmacy students for 10 consecutive days (24 h/day; preinterventional and post-interventional, respectively); development of a standardized data collection form in pilot phase; each medication dose observation could contain more than one error	Educational program consisting of an 1-h teaching session and 30-min individual practical training for preparation and administration of commonly used medications; education was repeated three times	Single	CG: 151 doses with ≥1 error/311 observed doses; IG: 87/284; clinical relevance was assessed by three experts (pharmacist, neonatologist, neonatological nurse); interventional effects addressed administration errors mainly	48.55%/30.63%	17.92% (significantly positive results)
Chua et al. (Malaysia, 2017) (58)	Drug administration errors	UBA, single center	Pediatric ward	Direct observation of drug administration by pharmacists on two pediatric wards for 40 days preintervention and post-intervention, respectively	Pharmacist-led presentation of preintervention drug administration errors to pediatric physicians and nurses, followed by discussion (repeated six times)	Single	Pre: 1,284 doses observed for 217 patients (5.9 doses per patient) with 569 doses containing at least one error (852 errors total); post: 1,401 doses for 208 patients (6.7 doses per patient) with 400 doses containing at least one error (496 errors total)	44.31%/28.55%	15.76% (significantly positive results)
Davis et al. (USA, 2017) (59)	Dispensing errors	UBA, single center	Hospital pharmacy	Implementation of an electronic work flow management system. The system interfaced with the computerized physician order entry	Electronic workflow management system	Single	Pre: 9.8 errors per 10,000 doses dispensed; post: 8.2 errors per 10,000 doses	0.098%/0.082%	0.016% (significantly positive results)
Marconi et al. (USA, 2012) (60)	Drug administration errors	UBA, single center	EP	Assessment of missed or delayed (>1 h later than scheduled time) medication administrations in the emergency department; separation of medications into “urgent” (medication for patients' chief complaint or acute diagnosis) and non-urgent medications	Emergency department pharmacist	Single	CG: 29 urgent and 169 non-urgent medications/723 medications; IG: 21 urgent and 64 non-urgent medications/851 medications	Missed or delayed medications total: 27.39%/9.99%	Missed or delayed medications total: 17.40% (significantly positive results)
Niemann et al. (Germany, 2015) (61)	Drug administration errors	UBA, single center	Pediatric ward	Direct observation by four trained pharmacists using a predefined 22-item list of drug-handling processes; monitoring in the morning (7:30–10:00 a.m.) for 20 working days	Three-step educational intervention: three-page handout (addressed knowledge deficits and memory-based lapses), 60-min pharmacist-led education (background information and drug-handling guidelines), and 56-page comprehensive reference book (detailed information about drug-handling)	Single	Patients: CG: 38/43 patients suffered ≥1 ME within the observed processes; IG: 25/51; processes: CG: 370/581 observed processes contained ≥1 error; IG: 100/441	Patients: 88.37%/49.02%; processes: 63.68%/22.68%	Patients: 39.35%; processes: 41.00% (significantly positive results)
Niemann et al. (Germany, 2014) (62)	Drug administration errors	UBA, single center	PICU	Direct observation by five trained pharmacists using a pre-defined 24-item-list of drug-handling processes; monitoring between 7:00–9:00 a.m. and 11:00 a.m. to 1:00 p.m. for 26 working days	Three-step educational intervention: three-page handout, 60-min pharmacist-led education, and 76-page comprehensive reference book	Single	Patients: CG: 36/38 patients suffered ≥1 ME within the observed processes; IG: 42/47; processes: CG: 384/668 observed processes contained ≥1 error; IG: 445/883	Patients: 94.74%/89.36%; processes: 57.49%/50.40%	Patients: 5.38%; processes: 7.09% (mixed results)
Ozkan et al. (Turkey, 2013) (63)	Drug administration errors	UBA, single center	Pediatric ward	Direct Observation of medication administration (observation period between 10:00 a.m. – 6:00 p.m. and 10:00 p.m. – 6:00 a.m.); assessment of a deviation between the physician's order and the administered medication	(1) Written alerts displayed on the door of the preparation room (2) Signaling arm bands for the medication preparing and administration nurses (3) Earlier medication delivery by pharmacy (4) Preparation and administration guidelines (5) Increase of nurse/patient-ratio	Bundle	CG: 475 errors/1,686 observed medication doses; IG: 313/1,460; workload determined as leading cause for administration errors (no significant difference between CG and IG)	28.17%/21.44%	6.73% (significantly positive results)
**Studies addressing multiple steps in the medication use process (combined medication errors)**									
Abuelsoud (Egypt, 2019) (64)	Combined medication errors (prescribing, drug administration, monitoring errors)	UBA, single center	Pediatric ward	FOCUS-PDCA technique (Plan-Do-Study-Act cycle); 100 medical files were randomly selected on a pediatric medical ward per month over a period of 9 months; it was aimed at reducing drug-related problems in each of the three selected steps of the medication use process prescribing, administration, and monitoring steps to ≤ 15% within 9 months	(1) Conducting an educational program to pediatric staff (physicians and nurses) (2) Implementation of a clinical pharmacist into the medical team (3) Establishment of drug information center (4) Establishment of IV admixture unit (5) Using auxiliary labels	Bundle	900 medical files reviewed (100 files per month); prescribing errors: CG (1st month): 47 errors/ 100 files, IG (9th month): 10 errors/ 100 files; drug administration errors: CG (1st month): 60 errors/ 100 files, IG (9th month): 10 errors/ 100 files; monitoring errors: CG (1st month): 56 errors/ 100 files, IG (9th month): 15 errors/ 100 files	Administration errors: 60%/10%; monitoring errors: 56%/15%	Administration errors: 50%; monitoring errors: 41% (significantly positive results)
Benkelfat et al. (Canada, 2013) (65)	Combined medication errors (prescribing errors, drug administration errors)	CCT, single center	EP	Retrospective ME analysis of medical records (drug choice, dosage deviation >10% of recommended dosing, frequency, and route of administration) for children <18 years, who were treated for anaphylaxis in the emergency department	Standard order form for medications used in anaphylaxis	Single	CG: 18 medical charts with ≥1 error/30 medical charts; IG: 16/29; dosing errors were significantly reduced, but not errors in total	60.00%/55.17%	4,83% (significantly positive results)
Ernst et al. (USA, 2017) (66)	Combined medication errors (prescribing errors, drug administration errors)	UBA, single center	NICU	A retrospective EMR chart review of children with a birth weight <2 kg and a hospitalization of ≥58 days were included in the time range of 2009–2013. The 2-month immunization status was investigated for the seven vaccines recommended	An electronic immunization alert was introduced into the EMR. It was shown from days 56 to 67 on the beginning of the day to the physicians and nurses separately	Single	CG: 35 infants partially immunized or unimmunized/121 infants; IG: 6 infants partially immunized or unimmunized/140 infants	29%/6%	23% (significantly positive results)
Fawaz et al. (Egypt, 2017) (67)	Combined medication errors (prescribing, transcribing, and drug administration errors)	UBA, single center	Operating room	Pharmacist observed drug handling in the operating room and reviewed prescribed medications	Pharmacist-led educational program consisting of detection, reporting, and prevention of medication errors	Single	Pre: 312 medication errors were detected in 110 patients with 936 medication orders (6.2 medication orders per patient); post: 224 medication errors in 112 patients with 693 medication orders (8.5 medication orders per patient)	33.33%/32.32%	1.01% (non-significant positive results)
Foster et al. (USA, 2013) (68)	Combined medication errors (prescribing errors, drug administration errors)	ITS, single center	EP	Ward pharmacist reviewed medication orders in the emergency department on weekdays from 3 to 11 pm; assessment of ME rates in three 3-month intervals	3-h educational program for emergency department residents, led by an attending physician and the ward pharmacist	Single	2 of 10 investigated drug-related problems showed significant improvements (dose adjustment and order clarification)	N/A	N/A (mixed results)
Keiffer et al. (USA, 2015) (69)	Combined medication errors (prescribing errors, drug administration errors)	UBA, single center	Pediatric cardiothoracic intensive care unit	Analysis of MEs that resulted in patient harm (NCC MERP type D-I) through assessment of voluntary reports by pharmacists, trigger tools, and hospital-wide voluntary incident reports by hospital-wide and unit-based quality leaders	(1) Quality process education (“the 5-rights”) (2) Nursing independent double check using a standardized checklist; (3) Hands-free communication with wearable voice-controlled devices (4) ME huddles (5) A “distraction-free zone” consisting of a physical mat in front of the PYXIS and signs placed on the computers in the unit (6) Bedside medication barcoding	Bundle	33 pADEs that resulted in patient harm in 2010; 3 pADEs in 2011; 6 pADEs in 2012; and 4 pADEs in 2013; harm-causing pADEs were reduced from 0.43 to 0.05 per 1,000 administered medication doses	N/A [88% error reduction from CG (2010) to IG (2013)]	N/A (significantly positive results)
Maaskant et al. (Netherlands, 2018) (70)	Combined medication errors (prescribing, drug administration, monitoring errors)	ITS, single center	PICU	Clinical records and the incident reporting system of a PICU were reviewed for medication errors. When an error was suspected, a pediatric intensivist, and a clinical pharmacist reviewed the case. Six timepoints preintervention and post-intervention, respectively	Pharmacist-led structured medication audit and feedback to pediatric intensivists on a PICU. A clinical pharmacist was present on ward 3 h 5 days per week. PICU-patients with “(a) reduced renal and/or hepatic clearance, (b) oncological diagnoses, (c) high-alert medication prescriptions, (d) receiving more than 5 medications, and (e) medication prescriptions with which the PICU professionals felt unfamiliar” were included.	Single	Pre: within 1 year, 254 patients were admitted to the PICU with 153 medication errors (2.27 medication errors per 100 prescriptions; 23 harmful medication errors); post: within 1 year, 230 patients were admitted with 90 medication errors (1.74 medication errors per 100 prescriptions; 6 harmful medication errors), 75 of these 230 patients were audited by the pharmacist: the prevalence of medication errors was found significantly lower in patients with medication audit	2.27%/1.74%	0.53% (significantly positive results)
Martin et al. (USA, 2017) (71)	Combined medication errors (prescribing, drug administration, monitoring errors)	UBA, single center	Operating room	Anesthesiologists were directly observed for 2 months regarding the drug handling and medication errors in the operating room. Using this data, a failure-mode-and-effect analysis was performed to develop interventions, followed by a reobservation	(1) Medication tray reorganization (pharmacy-prepared trays, reorganization with colors and sequesters; due to high-risk medications, and according to the frequency of usage) (2) Medication cart top template (standardized organization of common medications) (3) Syringe labeling (standard nomenclature and color-coding) (4) Infusion double check (independent double check, documented with preprinted labeling tape on the infusion) (5) Medication practice guideline (developed and posted in every operating room: syringe labeling, medication preparation)	Bundle	Pre: 368 syringes for 68 patients were audited with 101 labeling errors within 2 months; 17 infusion pumps were checked with 13-times double-check error. No standardized workspace organization was found; post: 402 syringes for 61 patients were audited with 16 labeling errors within 2 months. 17 infusion pumps were checked with 7-times double-check error	Labeling error: 27.4%/4.0%; infusion double check: 76.5%/41.2%	Labeling error: 23.5%; infusion double check: 35.3% (mixed results)
McClead et al. (USA, 2014) (72)	Combined medication errors (prescribing-, drug administration-, dispensing errors)	UBA, single center	Entire hospital	Within a pediatric hospital, a quality improvement initiative was implemented to reduce harm-causing medication errors (NCC MERP D-I) in a 4-year study period. The initiative rendered interventions to all aspects of the medication use process with a special focus on administration errors. Error data was recorded from voluntary incident reporting, trigger tool analysis, reversal agent review, and clinical pharmacist interventions	(1) Independent double-check policy for high-risk medications (2) Implementation of a wireless nurse communication system (3) Smart syringes and pumps with drug libraries (4) Safety nurse-led audits of the compliance to the 5 rights medication administration (5) Implementation of a barcoded medication administration (6) Pharmacy has more pneumatic tubes for faster delivery of compounded urgent medications	Bundle	In the 1st quarter 2010, the number of pADEs maximized to 85 within 3 months (0.171 pADE per 1000 dispensed doses). In the last investigated quarter (2nd quarter 2013), this number was reduced to 22 pADEs within 3 months (0.040 pADE per 1000 dispensed doses)	0.017%/0.004%	0.013% (significantly positive results)
Mekory et al. (Israel, 2017) (73)	Combined medication errors (prescribing errors, drug administration errors)	UBA, single center	Pediatric ward/emergency department	The Joint Commission International (JCI) accreditation in 2014 was sought. Therefore, a training program for the JCI standards to preclude prescribing and administration errors was implemented, and a prereview and post-review of handwritten emergency department and pediatric ward medical charts were performed	Educational program consisting of lectures, a personal handbook, and an educational software. Topics discussed were prescribing of an accurate order, filling it by the nurse, supervising it by the nurse and pharmacist, and handling of a medication error	Single	Pre: during 1 month, 183 patients were included, they got 937 prescription and 924 administration orders; 61 prescribing and 104 administration errors occurred; post: during 1 month, 183 patients were included; they got 961 prescription and 958 administration orders. 41 prescribing and 114 administration errors occurred	Administration errors: 11.3%/11.9%	Administration errors: −0.6% (non-significant negative results)
Migowa et al. (Kenya, 2018) (74)	Combined medication errors (prescribing and dispensing errors)	UBA, single center	EP, hospital pharmacy	Retrospective chart review of 1 year of prescriptions and medication dispensing records of physicians and pharmacists in an emergency department of a tertiary hospital	A voice recognition system was installed at one computer in the emergency department. A medical dictionary was developed and stored in a computer database; voice profiles were installed; training with the system was provided for the users	Single	Only duration of preinterventional and post-interventional phase submitted: 1 year; pre: 1,196 prescriptions were written for 1,196 patients with 889 errors. In the same period, 1,111 dispensations with 1,030 errors were documented; post: 501 prescriptions were written for 501 patients with 329 errors. In the same period, 356 dispensations with 332 errors were documented; most prescribing error reduction was seen in the dose prescription. pharmacists criticized that no drug database existed—this may have been contributed to the high error rate	Dispensing errors: 92.7%/93.3%	Dispensing errors: −0.5% (non-significant negative results)
Watts et al. (USA, 2013) (75)	Combined medication errors (prescribing, dispensing, administration errors)	UBA, single center	Oncology	Multidisciplinary chemotherapy safety team that met every 6 months to analyze all chemotherapy MEs and derive process optimizations	Optimizations included: (1) Routine order checking by pharmacist and administration nurse (2) Pharmacy standardization of drug dilutions	Bundle	2008: 33 chemotherapy errors/8,517 dispensed chemotherapy medications; 2009: 15/6,277; 2010: 23/9,523; 2011: 18/9,794; CG (2008): 3.9 errors/1,000 medications, IG (2011): 1.8/1,000	0.39%/0.18%	0.21% (significantly positive results)

*CCT, controlled clinical trial; CG, control group; EMR, electronic medical record; EP, emergency department; IG, intervention group; ITS, interrupted time–series study; ME, medication error; N/A, not available/not applicable; NICU, neonatal intensive care unit; pADE, preventable adverse drug event; PICU, pediatric intensive care unit; UBA, uncontrolled before–after study*.

### Synthesis of Results

Authors classified studies as either a “single-intervention study” or a “bundle-intervention study,” according to whether or not they implemented single or multiple interventions. The step(s) in the MUP targeted by the interventions were identified, and studies were also classified according to the errors they seek to reduce, either dispensing, drug administration, or monitoring errors individually or when they target multiple steps in the MUP as investigating “combined medication errors.” For combined medication error studies, the impact of interventions on MUP steps other than prescribing was extracted for this analysis.

**Figure 1:**
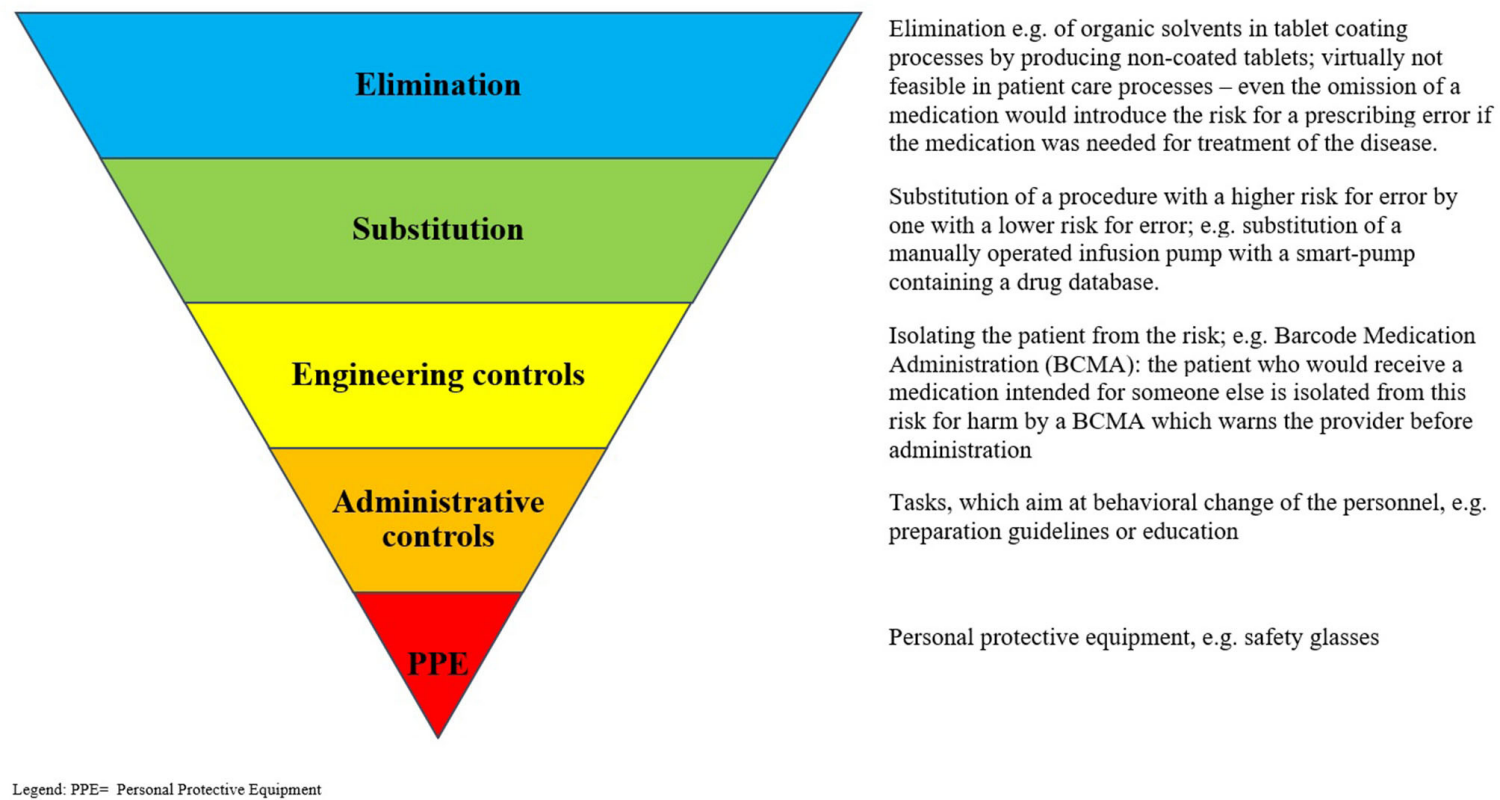
“Hierarchy of controls” with examples for each stage. PPE, Personal protective equipment. Adapted from: National Institute for Occupational Safety and Health (NIOSH) (76).

The individual interventions were classified according to a hierarchical approach to risk control (76). It has been suggested that this approach can aid in the identification of appropriate interventions to reduce known risks occurring during risk-prone processes, and it has been hypothesized that this approach could be successfully adopted in the healthcare setting (77). The highest (most likely to be successful) level of control involves eliminating the risk entirely; the next level suggests making a substitution, so that a risk is reduced when it is not possible to eliminate it (e.g., the substitution of manually operated infusion pumps with smart pumps containing a drug database). Engineering controls are next on the hierarchy and involve attempts to isolate a risk or to isolate a patient from a risk. For example, barcoded drug administration is an engineering control, which aims to reduce the risks of patients receiving the wrong medication via mandatory scanning a patient wristband and/or the medication to be given; if either is false, this is highlighted to the healthcare professional administering the medication, and they are prevented from proceeding to document the administration. This step is followed by administrative controls, which often include informative signage and education; personal protective equipment completes the hierarchical approach, as the lowest level of control. Figure 1 displays the “hierarchy of controls” with medication safety–related examples for each level. For analysis, elimination, substitution, and engineering controls were combined and considered as higher-level controls, to be compared with the lower levels of control (administrative controls and personal protective equipment). This division into higher- and lower-level controls was performed in analogy to the methods presented by Card et al. (78).

The classification of interventions was performed independently by two authors (JK, NY). Interrater agreement was calculated via weighted Cohen κ (52). Discrepancies were discussed and resolved by a third author (AE) when necessary. The interventions were then grouped descriptively to provide a practical overview of possible interventions at each control level.

Group comparisons were calculated using Fisher exact test (level of significance: *p* < 0.05) or Mann–Whitney *U*-test (two-tailed, *p* < 0.05), whatever was applicable.

Initially planned meta-analyses were deemed unfeasible because of heterogeneity in study designs, outcomes, the high number of uncontrolled before–after studies, and the high proportion of studies using a bundle of interventions, meaning the influence of one specific intervention cannot be quantified.

### Risk of Bias Across Studies

All studies meeting the inclusion criteria were included, regardless of whether they reported positive, neutral, or negative results. It was assumed that there was an equal distribution for each group in case of no bias.

It has been reported that positive study results are often published sooner than non-significant or negative results, producing a time-lag bias (79, 80). Within this review, time to publication was assessed for each study using the time from date of data completion to the date of electronic publication (81, 82). Studies were grouped according to their results: into studies with significantly positive results (studies which statistically significantly reduced error rates), non-significantly results (reduced or increased error rates observed, but statistical significance not demonstrated), or mixed results. No studies demonstrating significantly negative results (increased error rates) were identified. Statistical differences were calculated using Mann–Whitney *U*-test (see above).

### Additional Analyses

Definitions of medication errors were recorded and compared, following previous reports relating to heterogeneity of definitions (6, 11, 16, 83, 84), which can lead to poor comparability of studies. A definition was recorded when the authors of a study clearly identified that a definition had been used (e.g., “medication error was defined as…”). Authors searched all included studies for definitions of medication error, dispensing error, drug administration error, and monitoring error.

Whether or not the error types investigated in each study were clearly defined by the study authors was also assessed.

## Results

### Study Selection

**Figure 2:**
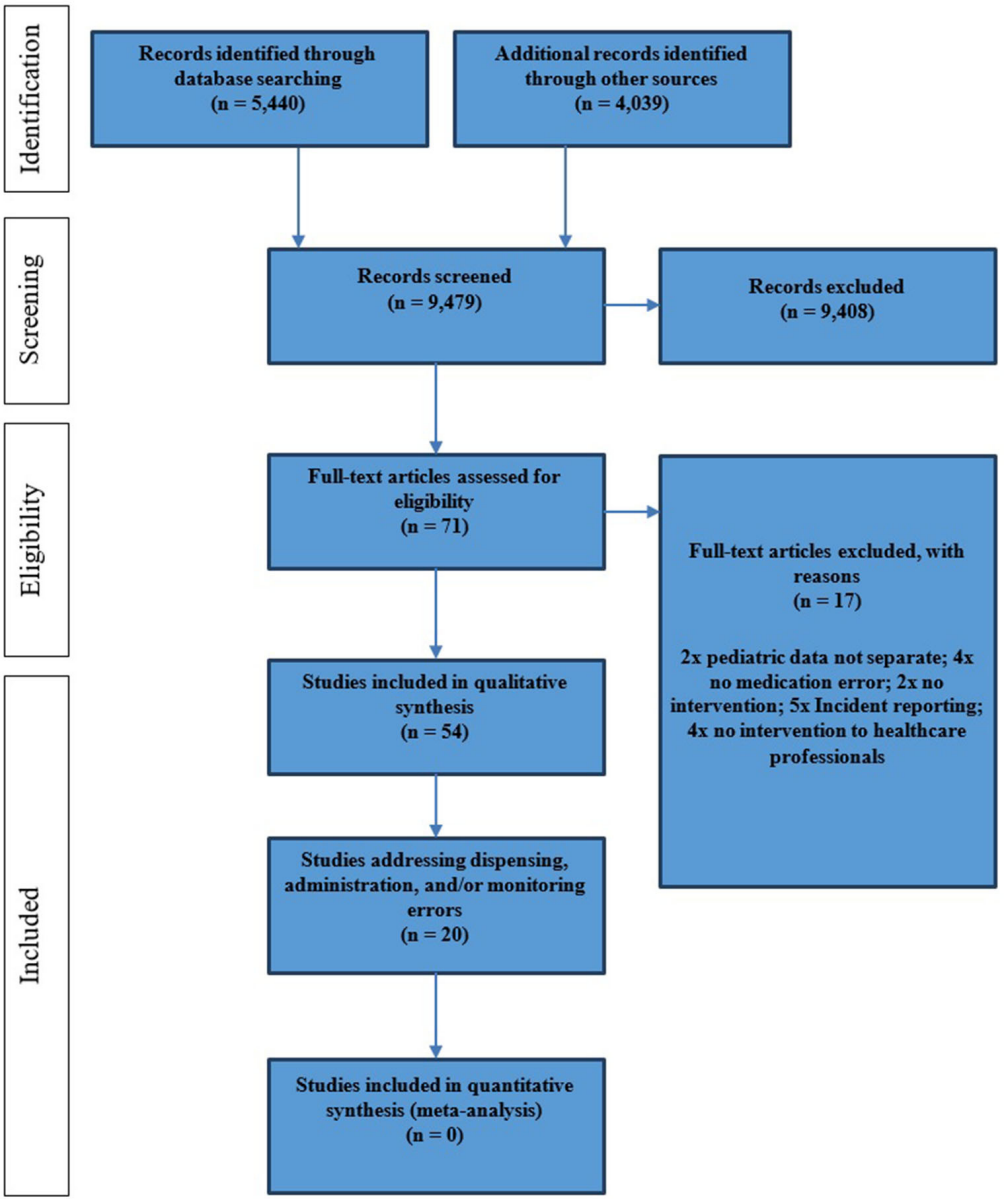
PRISMA flowchart reporting study selection.

The original search strategy was intended to identify studies investigating interventions to reduce pediatric medication errors during all stages of the MUP; the results were split *post-hoc* to address the challenge of managing dispensing, drug administration, and monitoring errors separately to prescribing errors. Database searches identified 5,440 abstracts, which were reviewed by the first reviewer (JK) and resulted in 50 full texts that corresponded to the full inclusion criteria. A second reviewer (AE) independently assessed 547 randomly selected abstracts. This resulted in an “excellent agreement” of both reviewers (Cohen κ = 0.86) (52). The search of the bibliographies of the included full texts as well as systematic and narrative reviews (*n* = 4,039 abstracts) led to four additionally included publications [see PRISMA flowchart (7), Figure 2]. Of the initial search results, 20 studies met the inclusion criteria for this review, in that they investigated interventions to reduce dispensing, drug administration, and/or monitoring errors.

### Study Characteristics

The 20 selected studies (56–75) originated from 11 countries and 4 continents (Table 2), with the majority being completed in North America (9/20, 45%). One controlled clinical trial and two interrupted time–series studies were identified; the majority of studies (*n* = 17; 85%) were classed as uncontrolled before–after studies. All studies addressed the hospital setting, with a focus on inpatient care (*n* = 15, 75%). Nineteen studies (95%) were single-center studies.

### Risk of Bias Within Studies

**Figure 3:**
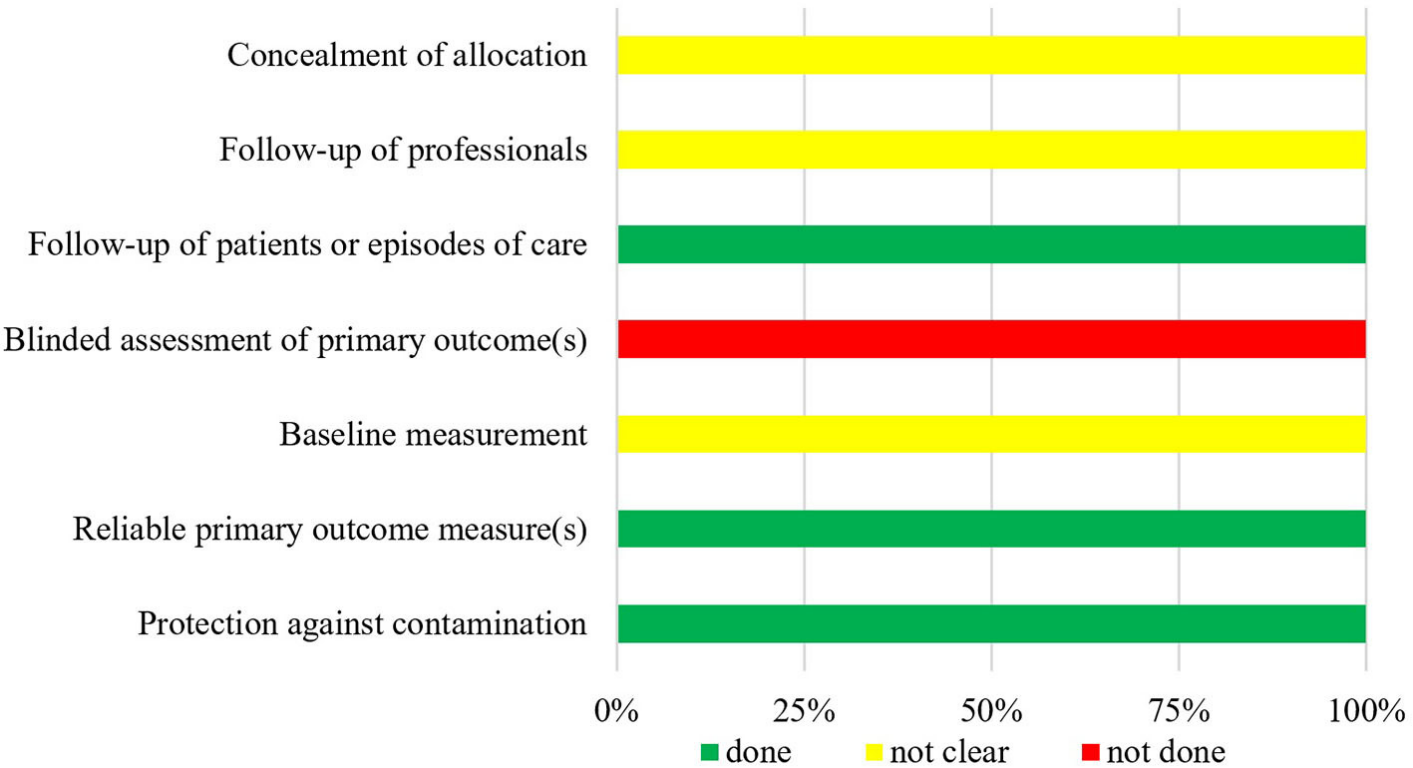
Bias risk assessment for included CCT (*n* = 1).

**Figure 4:**
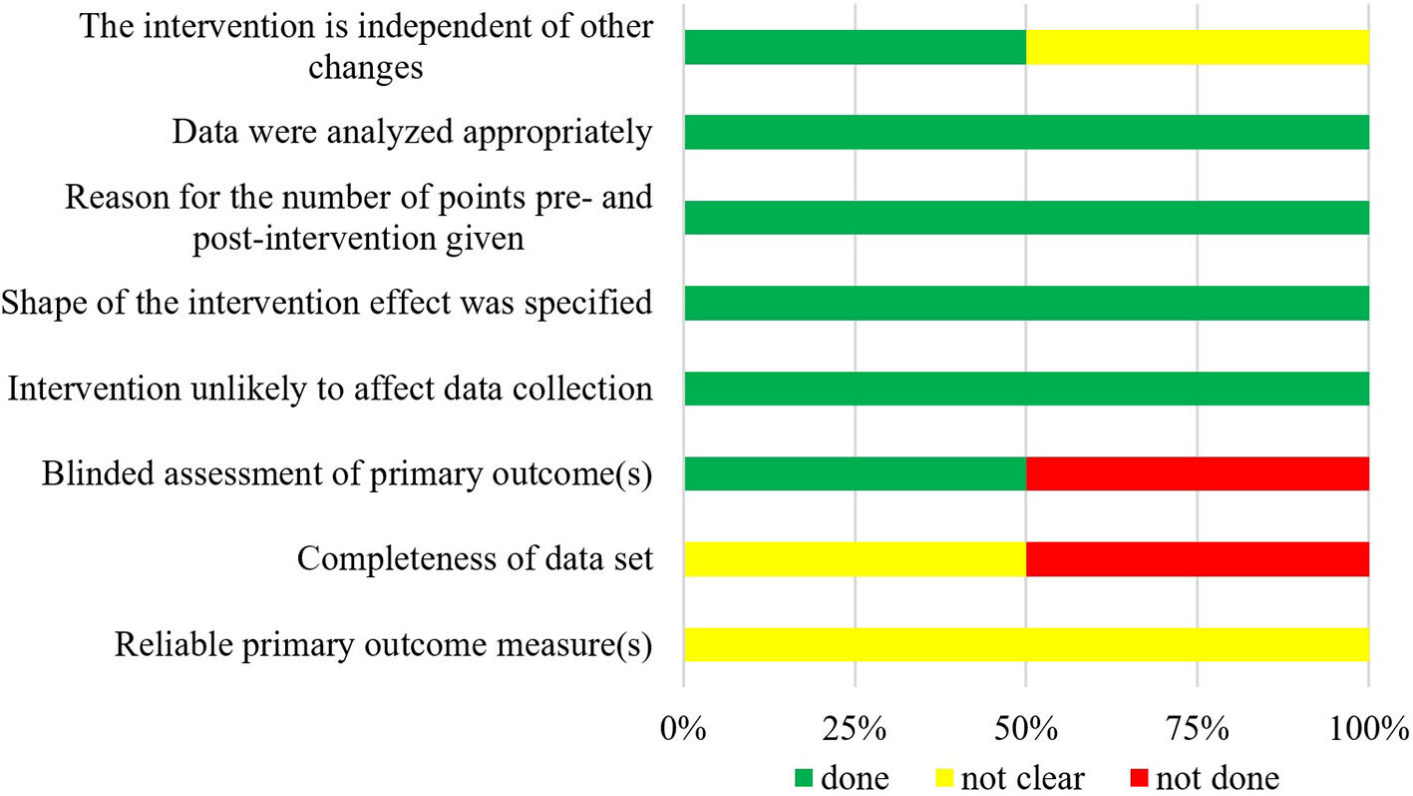
Bias risk assessment for included ITSs (*n* = 2).

**Figure 5:**
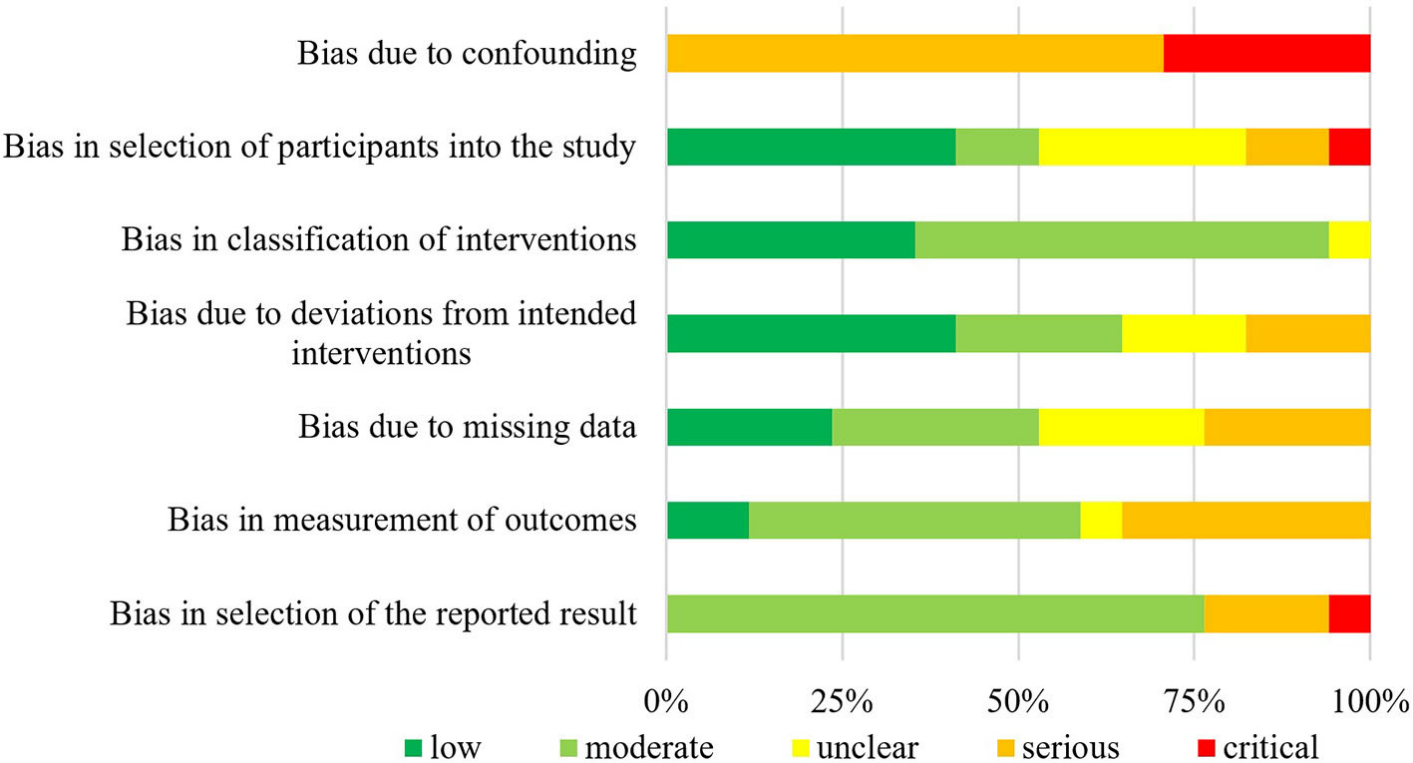
Bias risk assessment for included UBAs (*n* = 17).

The summarized data of bias risk assessment are shown in Figures 3–5 and Supplementary Tables 2–4. All uncontrolled before–after studies had a serious or critical risk for bias due to a non-declaration of possible confounders.

### Results of Individual Studies

The results of the individual studies are summarized in Table 2.

### Synthesis of Results

#### Interventions

A total of 44 different interventions were identified, which aim to reduce errors at one or more of the three included MUP stages. Eight of the included studies investigated interventions for a single point in the MUP, either dispensing or drug administration. No studies addressed monitoring errors independently of other MUP stages. The remaining 12 studies investigated interventions to reduce errors at more than one stage in the MUP and were therefore classified as combined medication error studies. The interventions in these studies, which were believed to address dispensing, drug administration, and/or monitoring, were extracted for descriptive analysis. Fourteen studies, including 34 interventions, achieved a statistically significant reduction in error rate, according to the definition of the individual study authors, 3 studies showed a statistically non-significant difference in error rates, and the remaining 3 studies reported mixed results.

#### Interventional Approach of the Studies

In the majority of studies (*n* = 13), the impact of a single intervention was investigated, whereas seven studies implemented a bundle of interventions. A non-statistically significant preference (*p* = 0.28) for a bundle of interventions was observed in studies targeting more than one stage of the MUP (42% of combined medication error studies used a bundle of interventions compared with 28% of studies investigating a single step in the MUP).

#### Single-Intervention Studies

Only one study investigated dispensing errors at pharmacy level, independent of other stages in the MUP. This study used a single intervention in the form of the implementation of a new electronic workflow system, which interfaced with the electronic prescribing system already in place and demonstrated a significant reduction in error rate (59).

Five of seven studies addressing drug administration errors implemented a single intervention. Marconi et al. investigated the impact of a clinical pharmacist working in an emergency department with the aim of reducing missed or delayed medications and demonstrated an absolute risk reduction of 17.4% (60). The remaining four single-intervention studies targeting drug administration errors implemented educational approaches: Chedoe et al. combined practical and theoretical preparation and administration techniques in one educational intervention for nurses on a neonatal intensive care unit (absolute risk reduction 17.9%) (57). Chua et al. used a pharmacist-led program involving observation of drug administration by the pharmacist followed by feedback and education to nursing and medical staff regarding observed risks or errors (absolute risk reduction 15.8%) (58). Niemann et al. implemented a similar educational program in two different settings; a short handout was combined with a lecture and a handbook for nurses on a pediatric ward and on a pediatric intensive care unit (absolute risk reduction 7.1 and 41.0%, respectively) (61, 62).

The single interventions implemented in the seven studies targeting multiple steps of the MUP included educational approaches (4/7), the use of computerized reminders, and the standardization of documentation (65–68, 70, 73, 74). Three of these reached significantly positive results (65, 66, 70).

#### Bundles of Interventions

Seven studies introduced a bundle of interventions, namely, more than one interventional method to address one or more stages in the MUP. Bundles of interventions were wide ranging and often included an element of education (56, 63, 64, 69, 71, 72, 75). The effect of a single intervention could not be calculated as the effects described in the studies were the result of the combination of interventions.

#### Classification of Interventions According to the Hierarchy of Controls

Two authors independently classified the 44 identified interventions according to the hierarchy of controls. Before discussion, agreement was 72% [weighted κ = 0.45, “fair” agreement (52)]. After discussion, complete consensus was reached.

**Figure 6:**
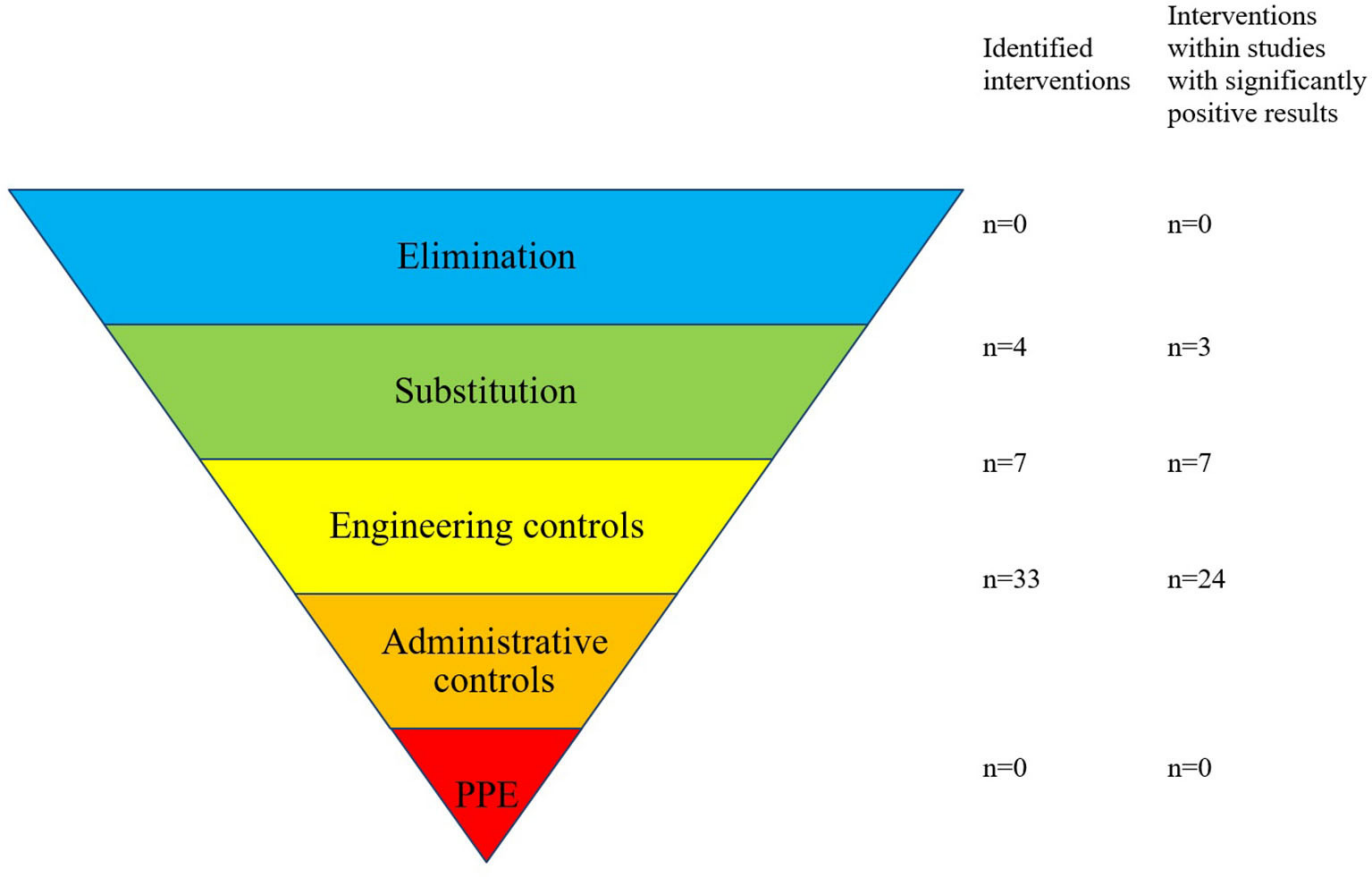
Forty-four interventions resulting from 20 studies were categorized to the “hierarchy of controls”. Adapted from: National Institute for Occupational Safety and Health (NIOSH) (76).

No studies were identified that eliminated the risk of error; in addition, only four interventions (9%) were rated as having made a substitution, for example, of equipment, with the aim of reducing the risk. Seven interventions (16%) were considered engineering controls, for example, the introduction of barcoded drug administration, whereas the vast majority of interventions were classified as “administrative controls” (33/44 interventions, 75%), such as educational programs, policies, guidelines, and warning signs (Figure 6).

Of the 44 identified interventions, 34 were implemented in the 14 studies that achieved a significant reduction in error rate. Three of the interventions involved in studies with reduced error rates were categorized as substitution controls, 7 as engineering controls, and 24 as administrative controls (Table 3).

**Table 3:** Summary of interventions that were implemented in studies that significantly reduced medication errors (34 interventions of total 44 identified interventions within 14 of 20 identified studies).

**Main risk control aspect**	**Number of interventions in studies with significantly positive results**	**Intervention type**
Substitution	*n* = 3	Standardized dilutions (75)
		Pharmacist production unit (64)
		Smart pumps (72)
Engineering controls	*n* = 7	Electronic workflow/CPOE (59)
		Enhanced medication delivery equipment (72)
		Hands-free communication equipment (2×) (69, 72)
		Barcoded medication administration (2×) (69, 72)
		Computerized alert (66)
Administrative controls	*n* = 24	Education and/or practical training (8×) (56–58, 61, 64, 69, 72)^*^
		Guidelines or protocols (6×) (56, 63, 65, 69, 72, 75)
		Rearrangement of staff or equipment (3×) (63, 64)^†^
		Expert consultation (4×) (60, 64, 70)^‡^
		Warning signs (3×) (63, 69)^§^

* *Keiffer et al. implemented two different educational interventions (medication error huddles and “5 rights education”)*.

† *Ozkan et al. implemented two different rearrangement interventions (decrease of patient-to-nurse ratio and modified delivery time of medications)*.

‡ *Abuelsoud et al. implemented two different expert consultation interventions (implementation of a clinical pharmacist within the medical team and a drug information service)*.

§ *Ozkan et al. implemented two different warning sign interventions (written alert on the door of the preparation room and signaling arm bands for medication-preparing nurses)*.

Six of the seven studies that implemented higher levels of control (substitution or engineering controls) resulted in a significant reduction in error rate (86%). In contrast, only 8 of 13 studies where solely administrative controls were implemented reported significant error reductions (62%). Thus, studies that implemented substitution or engineering controls were 1.4 times more likely to result in reduced error rates compared to administrative controls implemented alone (86:62 = 1.4). This difference failed to reach statistical significance (*p* = 0.23). Studies that implemented substitution or engineering controls lasted a median of 49 months to collect control- and intervention-group data, whereas studies of administrative controls had a median duration of 4 months. The difference between these median study durations was significant (Mann–Whitney, *p* < 0.05, two-tailed), demonstrating that the effects of administrative controls have been tested over shorter time periods, so the longevity of their effects is more difficult to establish.

Substitution controls included the introduction of a new pharmacy admixture unit, the introduction of smart pumps, and the use of standardized dilutions (64, 72, 75). These substitutions all formed part of successful bundles of interventions, which led to significantly reduced error rates in combined medication error studies. The introduction of voice-recorded and printed prescriptions did not lead to a reduced rate of dispensing errors (74).

Seven engineering controls were identified within four different studies (59, 66, 69, 72); five of these controls were implemented as parts of bundles of interventions, alongside administrative controls. They were demonstrated in all cases to be effective through a significant reduction in errors. Interventions included the introduction of a workflow management system, additional supplies to facilitate the use of pneumatic tubes, hands-free communication systems, and barcoded drug administration. Alerts in the patient electronic medical record were also successful on this occasion, although carefully implemented to reduce the risk of alert fatigue, which was acknowledged by the authors.

The overwhelming majority of interventions identified were classified as administrative controls. They could be subdivided into “education and training” (*n* = 12), “guidelines, protocols and procedures” (*n* = 8), “rearrangement of staff/material” (*n* = 6), “expert consultations” (*n* = 4), and “warning signs” (*n* = 3).

### Risk of Bias Across Studies

Based on the high percentage of significantly positive results (70%), a publication bias could not be ruled out.

Sixteen studies (80%) reported a date when data collection was completed, allowing the calculation of time to publication. Studies reporting significantly positive results were published after a median of 26 months (interquartile range = 17.5–37 months), whereas mixed results were published after a median of 39 months (interquartile range = 33.25–44.5 months). When compared to the distribution of significant positive results, a statistically significant difference could not be confirmed.

### Additional Analyses

In 14 of 20 full texts, 16 definitions were identified within 44 opportunities for definition (36%, see Supplementary Table 5). Seven definitions of a medication error (35% of all included full texts), one definition of a dispensing error (33% for a total of 3 full texts), seven definitions of a drug administration error (39%, total: 18 studies), and one definition of a monitoring error (33% for a total of 3 full texts) were identified. These definitions were heterogeneous in content and often failed to consider the patient; e.g., the definition for “medication error” contained only in one case the phrase “preventability”; the definitions for “drug administration error” did not contain any patient-centered aspect and had instead a technical focus.

## Discussion

### Summary of Evidence

We identified 44 individual interventions designed to reduce dispensing, drug administration, and monitoring errors and classified them according to a hierarchical approach to risk control. This is, to our knowledge, the first review to use the hierarchy of controls model to classify interventions for improving pediatric medication safety. This model, adapted from the health and safety industry, suggests that interventions should first be sought at the highest level of control (elimination of risk) before other options that are lower on the hierarchy and therefore potentially less effective are considered (78, 85). Although none of the interventions identified in this review were classified by the authors at the highest level (elimination), our results do support the theory that interventions at higher levels (substitution and engineering) should be prioritized, through the observation that studies implementing higher-level controls were 1.4 times more likely to achieve a significant reduction in error rates than those using administrative controls only. This is in line with the findings of Card et al., who reported a ratio of 1.6 (78). Despite this, we have observed that administrative controls are more frequently implemented, indicating a potential opportunity to rethink our approach to risk reduction and quality improvement, with a focus first on the opportunities to substitute risks or use engineering controls (77, 78), in favor of the perhaps easier-to-implement administrative controls.

There are criticisms of the use of a hierarchical control model in this setting. The lack of consideration of the potential human factors involved in medication errors has been cited as a key challenge (86), and Liberati et al. judged that “this model adds little value to the development of effective risk controls in clinical settings and lacks validity and usefulness” (87). The possibility of truly eliminating risks from the MUP can also be questioned, because omission of one or more medications would in itself be a risk to the patient (88). Practically seen, we may therefore expect interventions, which substitute a risk-prone process or step with a lower-risk option to be the strongest type of intervention available to us in the field of medication safety. We suggest that the hierarchy of controls model could be a useful tool to prompt those designing interventions to first consider the higher levels of control, before opting to implement administrative controls. We must, however, also acknowledge that the complexity of the MUP requires the use of a wider range of quality improvement tools and methodologies in order to design effective interventions, which also take local factors into consideration (85).

The wide range of interventions identified in this review at the higher levels of control supports this consideration. These interventions can be broadly considered as fitting into one of two groups. The first group includes interventions that interact with electronic medical records or electronic prescribing systems, which were already in place in the included studies (59, 66, 69, 72). On this basis, we would suggest that electronic prescribing and patient records provide a good foundation on which other interventions can be developed. The second group of higher-level interventions includes those that interact with the working environment, often with the aim of reducing interruptions and/or redistributing workload among the multidisciplinary team, for example, medication production being carried out by the pharmacy team in a specialized unit, rather than by the nursing team on the ward (64, 69, 72, 75). These improvements may lead to reduced error-producing conditions and in turn to improved pediatric medication safety (89).

The majority of the identified interventions were classified as administrative controls. Interestingly, we noted that these measures were often implemented alongside higher-level controls, as parts of bundles of interventions. In these studies, it is not possible to separate the influence of individual interventions within the bundle, and we must therefore acknowledge the potential importance of administrative controls including education, guidelines, and protocols when implemented alongside higher-level interventions.

In the review at hand, similar limitations of the currently published literature were identified as have been shown in previous systematic reviews in the field (6, 11, 34, 44). The included publications mainly originated from North America or Europe; the studies were performed primarily in inpatient settings, and the identified definitions of central terms were of heterogeneous nature. These factors should be considered, particularly when planning future research projects in this area.

## Strength and Limitations

We used the hierarchy of controls model for the first time to classify interventions to reduce pediatric dispensing, drug administration, and monitoring errors and have demonstrated that this model may be an appropriate tool for use in this setting.

Our methodology is potentially limited as the initial data extraction was performed by one researcher, with corrections and/or additions being provided by a second independent reviewer. As most studies were uncontrolled before–after studies and therefore present a high risk of bias, the results should be interpreted with caution. The inclusion criteria comprised studies published in English only, leading to a potential foreign language bias, although evidence for this is questionable (90). In addition, this review includes experimental studies for an 8-year time span only and must therefore be interpreted alongside the results of previous, and future subsequent, reviews on the same topic.

## Conclusion

When designing interventions to reduce pediatric dispensing, drug administration, and monitoring errors, the hierarchy of controls model should be considered, with a focus placed on the introduction of higher-level controls, which may be more likely to reduce errors than the administrative controls often seen in practice. A wide range of approaches to addressing the risks of dispensing and administering medications for pediatric patients in inpatient professional healthcare settings has been identified, and it is important to consider local conditions when planning interventions.

## Data Availability Statement

The original contributions presented in the study are included in the article/Supplementary Material, further inquiries can be directed to the corresponding author/s.

## Author Contributions

JK was responsible for search, data extraction/analysis, and drafted the first manuscript. NY assisted in the data analysis. UK, TO, and DB contributed to interpretation of data. AE developed the concept, supervised, and assisted with data extraction/analysis. JK, NY, UK, TO, DB, and AE revisited the manuscript critically for important intellectual content. They approved the final manuscript as submitted and take full responsibility for the manuscript. All authors are accountable for all aspects of the work and for ensuring that questions related to the accuracy or integrity of any part of the work are appropriately investigated and resolved.

## Conflict of Interest

The authors declare that the research was conducted in the absence of any commercial or financial relationships that could be construed as a potential conflict of interest.
