# Supplementary material for: Interventions to Reduce Medication Dispensing, Administration, and Monitoring Errors in Pediatric Professional Healthcare Settings: A Systematic Review
Source: Front Pediatr. 2021 May 26;9:633064. doi: 10.3389/fped.2021.633064 (PMC8187621; doi:10.3389/fped.2021.633064)
Supplement: Supplementary file 1 [file Data_Sheet_1.docx]

Supplementary Material

**Supplementary Material 1**: Detailed search strategies

**Medline**

#1 Child[mh] OR Infant[mh] OR "Infant, Newborn"[mh] OR Adolescent[mh] OR "Child, Preschool"[mh] OR child[tiab] OR infant[all] OR adolescent[all] OR children[all] OR infants[all] OR adolescents[all] OR "pediatric patient"[all] OR "pediatric patients"[all] OR adolescence[all] OR youth[all] OR youths[all] OR juvenile[all] OR childhood[all] OR teenager[all] OR teenagers[all] OR teen[all] OR teens[all] OR "preschool child"[all] OR neonate[all] OR newborn[all] OR baby[all] OR pediatric[tiab] OR pediatrics[tiab] OR paediatric[tiab] OR paediatrics[tiab]

#2 "Medication Errors"[mh] OR "medication error"[all] OR "medication errors"[all] OR "medications errors"[all] OR "medications error"[all] OR "medication error"[all] OR "prescribing error"[all] OR "prescribing errors"[all] OR "dosage error"[all] OR "dosage errors"[all] OR "dispensing error"[all] OR "dispensing errors"[all] OR "administration errors"[all] OR "administration error"[all] OR "drug verification error"[all] OR "drug verification errors"[all] OR "wrong drug administration"[all] OR "dosing error"[all] OR "dosing errors"[all] OR "dose error"[all] OR "dose errors"[all] OR "wrong dose"[all] OR "wrong dosage"[all] OR "wrong prescription"[all] OR "wrong drug"[all] OR "wrong medication"[all] OR "prescription error"[all] OR "prescription errors"[all] OR "medication safety"[all] OR "transcription error"[all] OR "transcription errors"[all] OR "transcribing error"[all] OR "transcribing errors"[all]

#3 #1 AND #2

#4 #3 AND 2011/11/22[pdat] : 2019/12/31[pdat]

**Embase**

1 exp child/ or exp infant/ or exp newborn/ or exp adolescent/ or exp preschool child/ or toddler.ti,ab. or child.ti,ab. or infant.mp. or adolescent.mp. or children.mp. or infants.mp. or adolescents.mp. or pediatric patient.mp. or pediatric patients.mp. or adolescence.mp. or youth.mp. or youths.mp. or juvenile.mp. or childhood.mp. or teenager.mp. or teenagers.mp. or teen.mp. or teens.mp. or preschool child.mp. or neonate.mp. or newborn.mp. or baby.mp. or pediatric.ti,ab. or pediatrics.ti,ab. or paediatric.ti,ab. or paediatric.ti,ab. [mp=title, abstract, heading word, drug trade name, original title, device manufacturer, drug manufacturer, device trade name, keyword]

2 exp medication errors/ or medication error.mp. or medication errors.mp. or medications errors.mp. or medications error.mp. or medication-error.mp. or prescribing error.mp. or prescribing errors.mp. or dosage error.mp. or dosage errors.mp. or dispensing error.mp. or dispensing errors.mp. or administration errors.mp. or administration error.mp. or drug verification error.mp. or drug verification errors.mp. or wrong drug administration.mp. or dosing error.mp. or dosing errors.mp. or dose error.mp. or dose errors.mp. or wrong dose.mp. or wrong dosage.mp. or wrong prescription.mp. or wrong drug.mp. or wrong medication.mp. or prescription error.mp. or prescription errors.mp. or medication safety.mp. or transcription error.mp. or transcription errors.mp. or transcribing error.mp. or transcribing errors.mp.

3 1 and 2

4 limit 3 to yr="2011 - 2019"

**Cinahl**

S1 MH “Child” OR MH “Infant” OR MH “Infant, Newborn” OR MH “Adolescence” OR MH “Child, Preschool” OR child OR infant OR adolescent OR children OR infants OR adolescents OR “pediatric patient” OR “pediatric patients” OR adolescence OR youth OR youths OR juvenile OR childhood OR teenager OR teenagers OR teen OR teens OR (preschool w1 child) OR neonate OR newborn OR baby OR pediatric OR pediatrics OR paediatric OR paediatrics

S2 MH “Medication Errors” OR medication w1 error OR medication w1 errors OR medications w1 errors OR medications w1 error OR medication-error OR prescribing w1 error OR prescribing w1 errors OR dosage w1 error OR dosage w1 errors OR dispensing w1 error OR dispensing w1 errors OR administration w1 errors OR administration w1 error OR drug w1 verification w1 error OR drug w1 verification w1 errors OR wrong w1 drug w1 administration OR dosing w1 error OR dosing w1 errors OR dose w1 error OR dose w1 errors OR wrong w1 dose OR wrong w1 dosage OR wrong w1 prescription OR wrong w1 drug OR wrong w1 medication OR prescription w1 error OR prescription w1 errors OR medication w1 safety OR transcription w1 error OR transcription w1 errors OR transcribing w1 error OR transcribing w1 errors

S3 S1 and S2

(limit to 11/01/2011 – 12/31/2019)

**CENTRAL**

#1 MeSH descriptor: [Child] explode all trees

#2 MeSH descriptor: [Infant] explode all trees

#3 MeSH descriptor: [Infant, Newborn] explode all trees

#4 MeSH descriptor: [Adolescent] explode all trees

#5 MeSH descriptor: [Child, Preschool] explode all trees

#6 "child":ti,ab,kw or "infant":ti,ab,kw or "adolescent":ti,ab,kw or "children":ti,ab,kw or "infants":ti,ab,kw or "adolescents":ti,ab,kw or "pediatric patient":ti,ab,kw or "pediatric patients":ti,ab,kw or "adolescence":ti,ab,kw or "youth":ti,ab,kw or "youths":ti,ab,kw or "juvenile":ti,ab,kw or "childhood":ti,ab,kw or "teenager":ti,ab,kw or "teenagers":ti,ab,kw or "teen":ti,ab,kw or "teens":ti,ab,kw or "preschool child":ti,ab,kw or "neonate":ti,ab,kw or "newborn":ti,ab,kw or "baby":ti,ab,kw or "pediatric":ti,ab,kw or "pediatrics":ti,ab,kw or "paediatric":ti,ab,kw or "paediatrics":ti,ab,kw

#7 MeSH descriptor: [Medication Errors] explode all trees

#8 "medication error":ti,ab,kw or "medication errors":ti,ab,kw or "medications errors":ti,ab,kw or "medications error":ti,ab,kw or "medication-error":ti,ab,kw or "prescribing error":ti,ab,kw or "prescribing errors":ti,ab,kw or "dosage error":ti,ab,kw or "dosage errors":ti,ab,kw or "dispensing error":ti,ab,kw or "dispensing errors":ti,ab,kw or "administration errors":ti,ab,kw or "administration error":ti,ab,kw or "drug verification error":ti,ab,kw or "drug verification errors":ti,ab,kw or "wrong drug administration":ti,ab,kw or "dosing error":ti,ab,kw or "dosing errors":ti,ab,kw or "dose error":ti,ab,kw or "dose errors":ti,ab,kw or "wrong dose":ti,ab,kw or "wrong dosage":ti,ab,kw or "wrong prescription":ti,ab,kw or "wrong drug":ti,ab,kw or "wrong medication":ti,ab,kw or "prescription error":ti,ab,kw or "prescription errors":ti,ab,kw or "medication safety":ti,ab,kw or "transcription error":ti,ab,kw or "transcription errors":ti,ab,kw or "transcribing error":ti,ab,kw or "transcribing errors":ti,ab,kw

#9 #1 or #2 or #3 or #4 or #5 or #6

#10 #7 or #8

#11 #9 and #10

#12 #11 Publication Year from 2011 to 2019

**Supplementary Table 1:** Exclusion criteria for abstract screening (adapted from Rinke *et al.*)

| **No.** | **Criteria** |
| --- | --- |
| (I) | No author listed and/or abstract not available |
| (II) | Full-text not published in English |
| (III) | Published prior to 2011/11/22 (Epub) |
| (IV) | Meeting/Conference abstract |
| (V) | Duplicate |
| (VI) | Case report only |
| (VII) | No original data |
| (VIII) | Does not include human data |
| (IX) | Does not include children |
| (X) | Does not provide pediatric data separately |
| (XI) | Does not include medication errors |
| (XII) | Does not include an intervention |
| (XIII) | Effort to reduce prescribing volume only |
| (XIV) | Simulation outcomes only |
| (XV) | Addresses misdiagnosis only |
| (XVI) | Other (specify) |

**Supplementary Table 2:** Bias risk assessment for the included Controlled Clinical Trial [n=1].

| **Controlled Clinical Trial** | Concealment of allocation | Follow-up of professionals | Follow-up of patients or episodes of care | Blinded assessment of primary outcome(s) | Baseline measurement | Reliable primary outcome measure(s) | Protection against contamination |
| --- | --- | --- | --- | --- | --- | --- | --- |
| Benkelfat R et al. (65) | **?** | **?** | **+** | **-** | **?** | **+** | **+** |
| Legend: “+”= done; “?”= not clear; “-”= not done. | | | | | | | |

**Supplementary Table 3:** Bias risk assessment for included Interrupted Time Series Studies [n=2].

| **Interrupted Time Series Studies** | The intervention is independent of other changes | Data were analyzed appropriately | Reason for the number of points pre- and post-intervention given | Shape of the intervention effect was specified | Intervention unlikely to affect data collection | Blinded assessment of primary outcome(s) | Completeness of data set | Reliable primary outcome measure(s) |
| --- | --- | --- | --- | --- | --- | --- | --- | --- |
| Foster ME et al. (68) | **?** | **+** | **+** | **+** | **+** | **-** | **-** | **?** |
| Maaskant JM et al. (70) | **+** | **+** | **+** | **+** | **+** | **+** | **?** | **?** |
| Legend: “+”= done; “?”= not clear; “-”= not done. | | | | | | | | |

**Supplementary Table 4:** Bias risk assessment for included Uncontrolled Before-After Studies [n=17].

| **Uncontrolled Before-After Studies** | Bias due to confounding | Bias in selection of participants into the study | Bias in classification of interventions | Bias due to deviations from intended interventions | Bias due to missing data | Bias in measurement of outcomes | Bias in selection of the reported result |
| --- | --- | --- | --- | --- | --- | --- | --- |
| Abuelsoud N et al. (64) | **-** | **?** | **+** | **+** | **?** | **-** | **-** |
| Campino A et al. (56) | **-** | **++** | **++** | **++** | **+** | **++** | **+** |
| Chedoe I et al. (57) | - | -- | ++ | ++ | - | + | + |
| Chua SS et al. (58) | **-** | **++** | **++** | **-** | **-** | **+** | **+** |
| Davis SJ et al. (59) | -- | ? | ? | ++ | ? | ? | -- |
| Ernst KD (66) | **-** | **++** | **+** | **++** | **++** | **++** | **+** |
| Fawaz MG et al. (67) | **-** | **++** | **+** | **++** | **+** | **+** | **+** |
| Keiffer S et al. (69) | **--** | **-** | **+** | **?** | **?** | **-** | **-** |
| Marconi GP et al. (60) | - | + | + | - | ++ | + | + |
| Martin LD et al. (71) | - | ++ | ++ | ++ | + | - | - |
| McClead RE et al. (72) | **--** | **?** | **?** | **?** | **-** | **-** | **+** |
| Mekory M et al. (73) | **-** | **++** | **+** | **++** | **++** | **+** | **+** |
| Migowa AN et al. (74) | **-** | **+** | **+** | **+** | **+** | **-** | **+** |
| Niemann D et al. (61) | - | ++ | ++ | + | ++ | + | + |
| Niemann D et al. (62) | - | ? | + | + | ? | + | + |
| Ozkan S et al. (63) | -- | - | + | - | + | - | + |
| Watts RG et al. (75) | **--** | **?** | **+** | **?** | **-** | **+** | **+** |
| Legend: “++”, low risk of bias; “+”, moderate risk of bias; “-”, serious risk of bias; “--”, critical risk of bias; “?”, unclear risk of bias. | | | | | | | |

**Supplementary Table 5:** Definitions for Medication Error, Dispensing-, Drug Administration and Monitoring Error reported in the included full-texts.

| **First author** | **Design** | **Error type** | **Medication Error** | **Dispensing Error** | **Administration Error** | **Monitoring Error** | **Total definitions** | **Opportunities for definition** |
| --- | --- | --- | --- | --- | --- | --- | --- | --- |
| Abuelsoud N (64) | UBA | Combined medication errors | N/A | N/A | "any error occurred during drug administration (wrong intravenous (IV) diluent, IV admixture concentration or IV infusion rate, or wrong instructions for use)" | "any error occurred due to lack of drug monitoring (monitoring serum levels for anti-epileptics and aminoglycosides, vancomycin, or monitoring of specific drug parameters)" | 2 | 3 |
| Benkelfat R et al. (65) | CCT | Combined medication errors | Citing Walsh et al., a “medication error may be defined as an error in drug ordering, transcribing, dispensing, administration or monitoring”. | N/A | N/A | N/A | 1 | 2 |
| Campino A et al. (56) | UBA | Administration errors | N/A | N/A | N/A | N/A | 0 | 2 |
| Chedoe I et al. (57) | UBA | Administration errors | N/A | N/A | "A medication error was defined as any deviation in preparation or administration of the medication or both from the doctor’s prescription, the hospital’s intravenous policy or the manufacturer’s instructions" | N/A | 1 | 2 |
| Chua SS et al. (58) | UBA | Administration errors | N/A | N/A | Citing Chua SS et al. and Greengold NL et al., a "drug administration error was defined as a discrepancy between the drug regimen received by the patient and that intended by the prescriber and also drug administration procedures that did not follow standard hospital policies and procedures." | N/A | 1 | 2 |
| Davis SJ et al. (59) | UBA | Dispensing errors | N/A | N/A | N/A | N/A | 0 | 2 |
| Ernst KD et al. (66) | UBA | Combined medication errors | N/A | N/A | N/A | N/A | 0 | 2 |
| Fawaz MG et al. (67) | UBA | Combined medication errors | "A medication error is any error in the medication use process, whether it resulted in adverse outcomes or not." | N/A | N/A | N/A | 1 | 2 |
| Foster ME et al. (68) | ITS | Combined medication errors | N/A | N/A | N/A | N/A | 0 | 2 |
| Keiffer S et al. (69) | UBA | Combined medication errors | A “Medication Error is defined as an error that occurs with the prescribing, transcribing, dispensing, administration, adherence, or monitoring of a drug regardless of whether it results in patient harm or has the potential to result in patient harm”. | N/A | N/A | N/A | 1 | 2 |
| Maaskant JM et al. (70) | ITS | Combined medication errors | Citing NCC MERP, "any preventable event that may cause or lead to inappropriate medication use or patient harm while the medication is in the control of the health care professional, patient or consumer. Such events may be related to professional practice, health-care products, procedures and systems, including prescribing; order communication; product labeling; packaging and nomenclature; compounding; dispensing; distribution; administration; education; monitoring and use." | N/A | N/A | N/A | 1 | 3 |
| Marconi GP et al. (60) | UBA | Administration errors | N/A | N/A | N/A | N/A | 0 | 2 |
| Martin LD et al. (71) | UBA | Combined medication errors | N/A | N/A | N/A | N/A | 0 | 3 |
| McClead RE et al. (72) | UBA | Combined medication errors | "Medications errors are mistakes in the prescribing, dispensing, administration, or monitoring of medications." | N/A | N/A | N/A | 1 | 3 |
| Mekory TM et al. (73) | UBA | Combined medication errors | N/A | N/A | Medication administration error (MAE) were defined as any inconsistency, whether in drug, dosage form, dose, administration route, dosing interval, between what has been ordered by a physician and what the patient receives by nurse (as appears on nurse’s charts). | N/A | 1 | 2 |
| Migowa AN et al. (74) | UBA | Combined medication errors | N/A | “A dispensing error was defined as a discrepancy between a correct prescription and the actual medication instructions that the dispensing pharmacist issued to the patient.” | N/A | N/A | 1 | 2 |
| Niemann D et al. (61) | UBA | Administration errors | Citing Ferner et al., Medication Errors were “defined as failure in the treatment process that results in, or has the potential of resulting in, harm to the patient”. | N/A | "medication errors in drug-handling processes as defined by any deviation from internal and external drug preparation/ administration guidelines, the corresponding summaries of product characteristics or manufacturers’ recommendations." | N/A | 2 | 2 |
| Niemann D et al. (62) | UBA | Administration errors | N/A | N/A | "We aimed to identify and prevent medication errors in drug handling, including processes of drug preparation and administration performed by nurses in daily routine" | N/A | 1 | 2 |
| Ozkan S et al. (63) | UBA | Administration errors | N/A | N/A | "In this study, a difference between the physician’s order and the drug given to the patient was considered medication administration error. [...] a deviation of one hour from the time when the drug should be given to the patient was considered time error." | N/A | 1 | 2 |
| Watts RG et al. (75) | UBA | Combined medication errors | Citing the Committee on Quality of Health Care in America, “Medication errors were broadly defined using the definition suggested by the Institute of Medicine as any error in the medication use process to include prescribing, transcribing, dispensing, administration, or monitoring”. | N/A | N/A | N/A | 1 | 2 |
| Legend: CCT= controlled clinical trial; ITS= interrupted time-series study; UBA= uncontrolled before-and-after study; N/A= not applicable. | | | | | | | | |
